# Targeting oxidative stress in diabetic retinopathy: mechanisms, pathology, and novel treatment approaches

**DOI:** 10.3389/fimmu.2025.1571576

**Published:** 2025-06-16

**Authors:** Wei He, Panli Tang, Hongbin Lv

**Affiliations:** ^1^ Department of Ophthalmology, Taikang Sichuan Hospital, Chengdu, China; ^2^ Department of Cardiothoracic Surgery, The General Hospital of Western Theater Command, Chengdu, China; ^3^ Department of Ophthalmology, Affiliated Hospital of Southwest Medical University, Luzhou, China

**Keywords:** diabetic retinopathy, oxidative stress, reactive oxygen species, mitochondrial dysfunction, therapeutic strategies

## Abstract

Diabetic retinopathy (DR) is a common and severe microvascular complication of diabetes, leading to vision impairment and blindness, particularly in working-age adults. Oxidative stress plays a central role in the pathogenesis of DR, with excessive reactive oxygen species (ROS) damaging retinal tissues, including blood vessels and neurons. This oxidative damage is exacerbated through various metabolic pathways, such as the polyol pathway, protein kinase C(PKC) activation, and advanced glycation end-product(AGE) formation. Additionally, mitochondrial dysfunction, retinal cell apoptosis, inflammation, and lipid peroxidation are key pathological processes associated with oxidative stress in DR. Epigenetic modifications, including DNA methylation and histone alterations, further contribute to gene expression changes induced by oxidative stress. To mitigate oxidative damage, therapeutic strategies targeting ROS production, neutralizing free radicals, and enhancing antioxidant defenses hold promise. Various natural antioxidant compounds, such as polyphenols (e.g., epigallocatechin-3-gallate, quercetin, resveratrol) and carotenoids (e.g., lutein, zeaxanthin), have demonstrated potential in reducing oxidative stress and improving retinal health in DR models. Moreover, activation of the Nrf2 and SIRT1 pathways has emerged as a promising approach to enhance the antioxidant response. Although preclinical studies show promising results, further clinical trials are necessary to validate the efficacy and safety of these therapeutic strategies. In conclusion, a better understanding of the molecular mechanisms underlying oxidative stress in DR and the development of multi-target therapies could provide more effective treatment options for DR patients.

## 1 Introduction

Diabetes mellitus (DM) has become one of the most severe global health challenges of the 21st century, with its complications posing a significant threat to human health (1). Among these, DR stands out as one of the most common microvascular complications and a leading cause of vision impairment in both working-age and older adults (2).

Chronic hyperglycemia-induced oxidative stress plays a pivotal role in the onset and progression of DR, as excessive ROS, primarily generated under high glucose conditions, inflict damage on tissues surrounding retinal blood vessels and neurons, thereby driving DR pathogenesis (3). Research indicates that oxidative damage in the retina induced by hyperglycemia is mediated through four key metabolic disturbances: activation of the protein kinase C (PKC) pathway, flux through the polyol pathway, activation of the hexosamine pathway, and intracellular formation of advanced glycation end products (AGEs) (4).

In addition to these metabolic disruptions, aberrant epigenetic modifications (5), altered nuclear factor activity, and mitochondrial dysfunction induced by hyperglycemia (6), all contribute to the excessive generation of ROS in DR. Notably, oxidative stress driven by epigenetic modifications can persist long after blood glucose levels normalize, a phenomenon referred to as “metabolic memory” (7). Furthermore, hyperglycemia-induced oxidative stress leads to mitochondrial dysfunction, cell apoptosis, inflammation, lipid peroxidation, and structural and functional changes in the retina, all of which contribute to DR pathology. Consequently, therapeutic approaches targeting DR have focused on reducing ROS production and enhancing ROS clearance via these pathways.

Oxidative stress has been increasingly recognized as a central driver of DR, mediating a wide array of pathological changes across retinal cell types (8). Numerous studies have detailed the role of ROS, mitochondrial dysfunction, and inflammation in DR progression. However, a unified perspective integrating oxidative stress with epigenetic regulation, immune dysregulation, and therapeutic advances remains lacking (9).

In this review, we aim to provide a comprehensive and updated synthesis of the mechanisms by which oxidative stress contributes to DR, with particular emphasis on molecular pathways, cell-specific vulnerability, and emerging antioxidant strategies, including nutraceuticals and targeted delivery systems. Although DR is a multifactorial disease involving inflammation, neurodegeneration, and metabolic dysregulation, hyperglycemia remains a central trigger for oxidative stress in the diabetic retina (10). This review specifically focuses on the oxidative stress mechanisms induced by hyperglycemia and their downstream effects in the pathogenesis of DR. By combining insights from basic science and translational studies, we hope to offer novel perspectives that may inform future therapeutic development.

## 2 Oxidative stress and ROS in DR

In the hyperglycemic state characteristic of diabetes, oxidative stress arises from an imbalance between free radical production and antioxidant defense mechanisms (11). This hyperglycemia-driven oxidative stress plays a pivotal role in damaging neuronal, vascular, and retinal tissues, and is central to the development and progression of DR (8).

Oxidative stress is defined as an imbalance between the production and clearance of ROS, which are highly reactive free radicals derived from molecular oxygen (12). In the diabetic retina, chronic hyperglycemia is the primary driver of excessive ROS generation, contributing to cumulative damage in vascular, neuronal, and glial cells. The retina is particularly susceptible to oxidative stress due to its high metabolic demand and abundant mitochondrial content (13).

**Figure 1:**
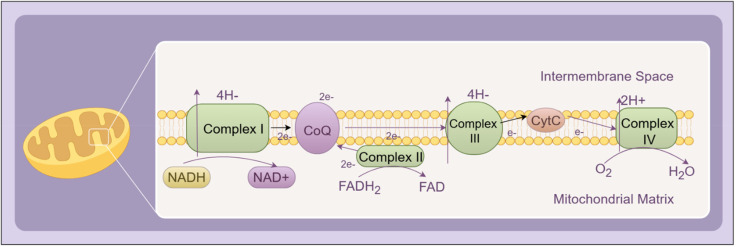
Schematic representation of the mitochondrial electron transport chain (ETC) under physiological conditions. Electrons from NADH and FADH_2_ are transferred through Complexes I–IV, ultimately reducing molecular oxygen to water. The proton gradient generated across the inner mitochondrial membrane drives ATP synthesis via ATP synthase. Complexes I–IV collectively constitute the core components of the ETC.

Mitochondria represent the predominant endogenous source of ROS. Under physiological conditions, the mitochondrial electron transport chain (ETC) transfers electrons from NADH and FADH_2_ to oxygen, ultimately producing water through oxidative phosphorylation (14). However, in hyperglycemic conditions, excess substrate influx leads to overproduction of reducing equivalents (NADH/FADH_2_), elevating the mitochondrial membrane potential and promoting electron leakage—particularly at complexes I and III—resulting in superoxide (O_2_•^–^) formation. This process becomes a significant initiator of oxidative stress in DR (15). A schematic representation of this classical ROS generation mechanism through the mitochondrial ETC is shown in **Figure 1**.

Recent research has substantially expanded our understanding of mitochondrial involvement in DR beyond classical ETC electron leakage. Studies have shown that hyperglycemia-induced mitochondrial fragmentation, mediated by an imbalance in fission and fusion proteins such as Drp1 and OPA1, contributes to mitochondrial dysfunction and impairs ATP synthesis in retinal capillary and Müller cells (16). In addition, defective mitophagy, the selective removal of damaged mitochondria, exacerbates ROS accumulation and promotes chronic stress signaling (17).

**Figure 2:**
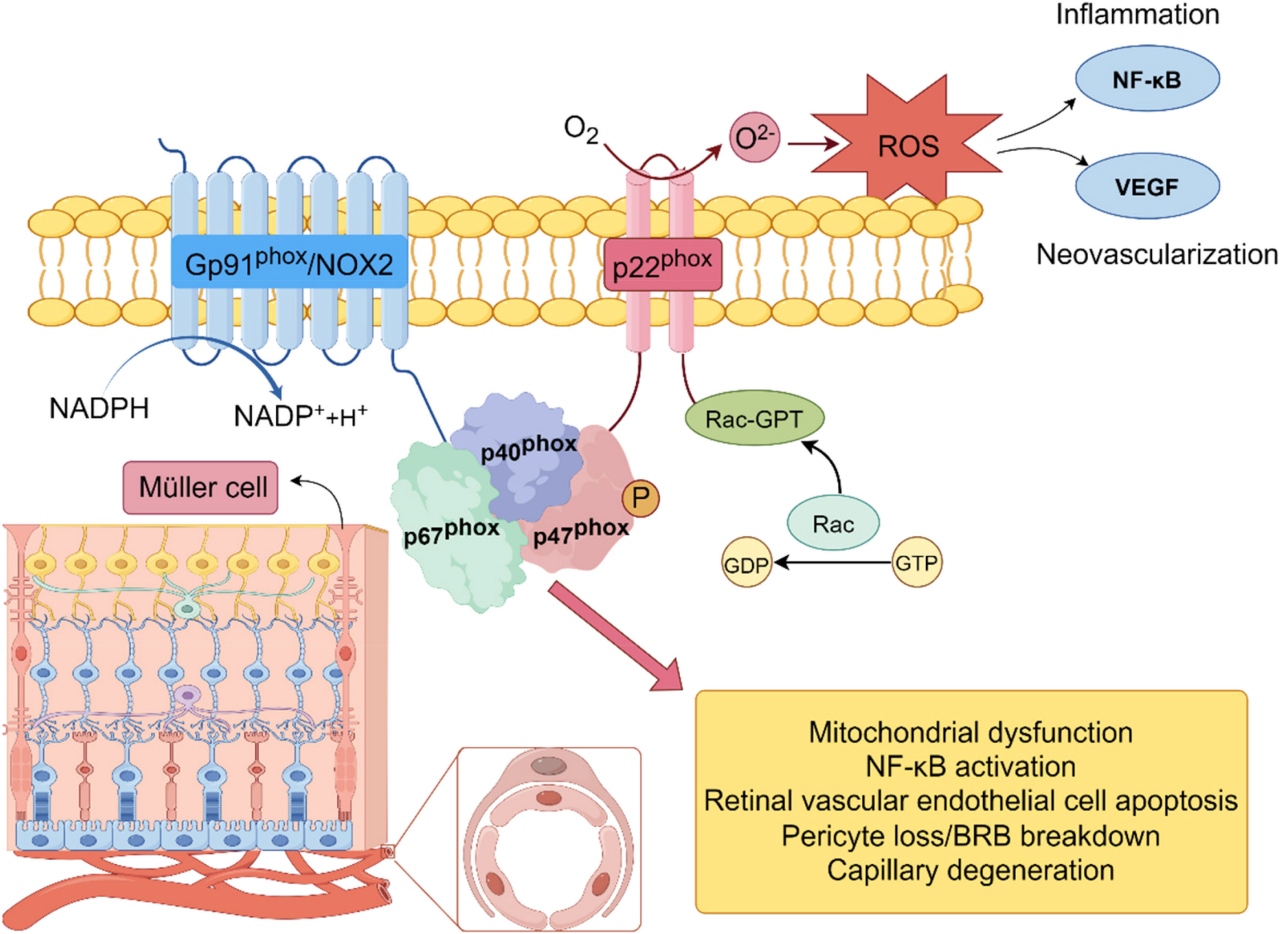
Mechanism of superoxide anion (O_2_^–^) production by NADPH oxidase (Nox2) in the cellular membrane. NADPH oxidase complex (Nox2), consisting of subunits Gp91phox, p22phox, p47phox, and p67phox, is activated by Rac-GTP. Upon activation, the complex transfers electrons from NADPH to molecular oxygen (O_2_), leading to the production of superoxide anion (O_2_^–^). This process is a primary source of reactive oxygen species (ROS) and plays a key role in the induction of oxidative stress.

Furthermore, NADPH oxidase 2 (Nox2)-mediated cytosolic ROS has been shown to stimulate mitochondrial dysfunction in a feedforward loop, intensifying mitochondrial ROS generation. Epigenetic modifications, such as mtDNA hypermethylation, impair mitochondrial gene expression and respiration, while decreased SIRT3 activity contributes to the loss of redox homeostasis in retinal endothelial cells (18). These findings emphasize that oxidative phosphorylation is not only disrupted by hyperglycemia but also acts as a sustained source of ROS that drives retinal cell injury. **Figure 2** illustrates recent findings regarding mitochondrial structural and functional dysregulation under hyperglycemic stress, including fission/fusion imbalance, impaired mitophagy, and persistent ROS amplification.

Collectively, the emerging evidence highlights that mitochondria are both a source and a target of oxidative stress in DR. The convergence of metabolic overload, mitochondrial fragmentation, and impaired antioxidant regulation creates a vicious cycle of ROS generation and mitochondrial dysfunction, contributing to apoptosis, inflammation, and neovascularization in the diabetic retina.

### 2.1 Metabolic memory in DR

While the role of oxidative stress in the pathogenesis of DR is well-established, recent studies suggest that the long-term effects of hyperglycemia—known as ‘metabolic memory’—also play a critical role in disease progression (19). This phenomenon involves persistent molecular alterations that continue to affect retinal cells even after blood glucose levels are normalized. Metabolic memory refers to the phenomenon in which prolonged exposure to hyperglycemia leads to persistent molecular alterations, even after blood glucose levels are normalized (20). This concept is critical in understanding the long-term effects of diabetes on the retina, particularly in DR. Although blood glucose control is crucial in preventing the development of DR, recent studies have shown that even after achieving normoglycemia, the retina may continue to experience oxidative stress, inflammation, and mitochondrial dysfunction due to epigenetic changes induced by prior hyperglycemic episodes (21).

These epigenetic alterations, such as DNA methylation and histone modifications, have been linked to the dysregulation of key oxidative stress-related genes, thereby perpetuating retinal damage (22). In particular, hyperglycemia-induced metabolic memory leads to long-lasting alterations in mitochondrial DNA, contributing to impaired mitochondrial function and the excessive generation of ROS. These persistent effects of metabolic memory are thought to exacerbate the pathological processes of DR, including retinal cell apoptosis and neovascularization. Recent studies have underscored the importance of targeting epigenetic modifications in the management of DR, offering new therapeutic avenues to reverse or mitigate the effects of metabolic memory (23). For instance, interventions aimed at restoring the function of antioxidant enzymes, such as superoxide dismutase 2 (SOD2), have shown promise in protecting retinal cells from the long-term damage caused by hyperglycemia-induced metabolic memory (24).

## 3 Metabolic abnormalities associated with oxidative stress in DR

**Figure 3:**
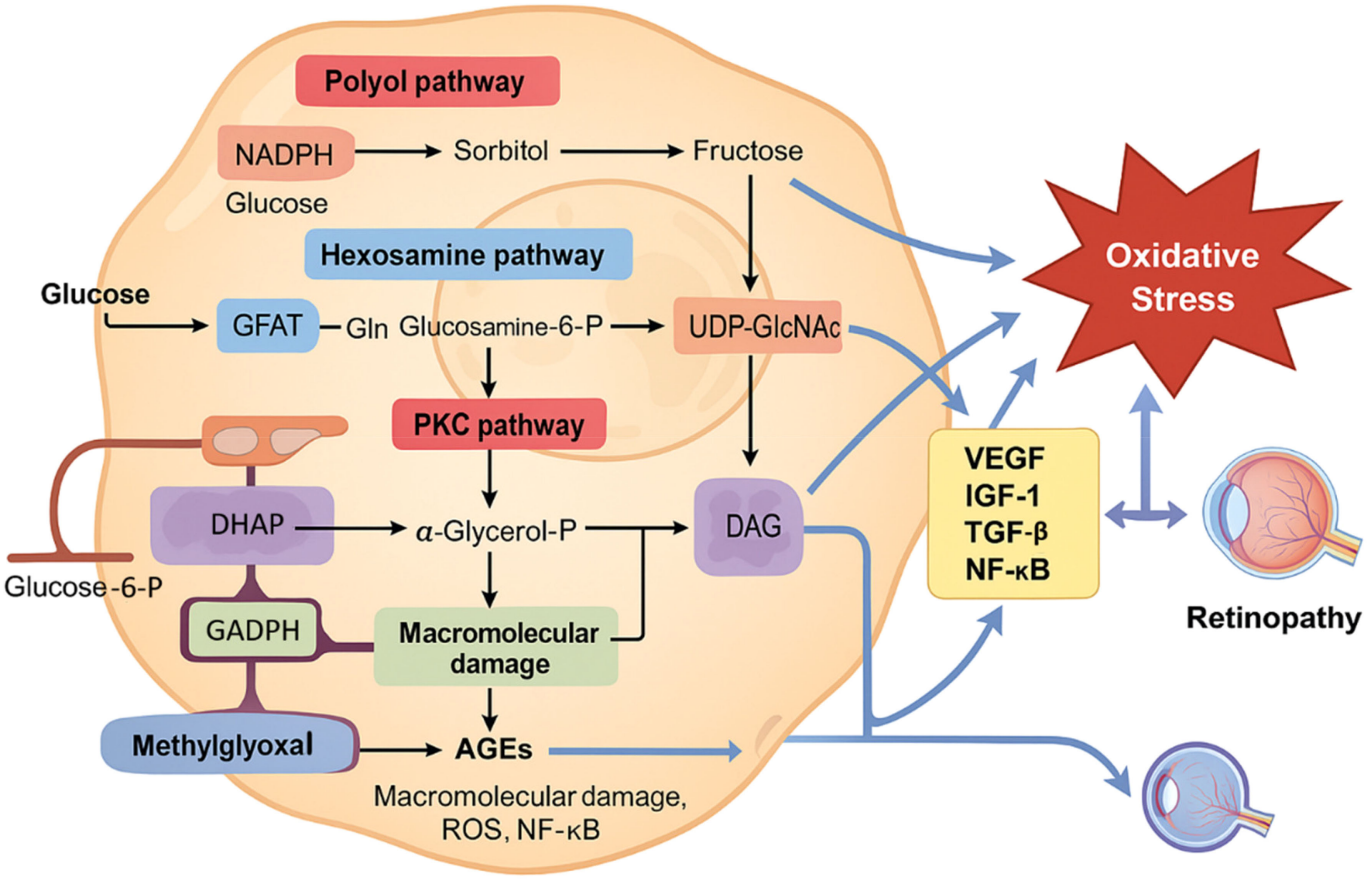
Metabolic disturbances in hyperglycemia contribute to oxidative stress and diabetic retinopathy. Chronic hyperglycemia activates multiple metabolic pathways, including the polyol pathway, the hexosamine biosynthesis pathway, protein kinase C (PKC) activation, and advanced glycation end product (AGE) formation. These interconnected pathways lead to increased generation of reactive oxygen species (ROS), primarily through mitochondrial dysfunction and NADPH oxidase activity. Excess ROS induces damage to retinal endothelial cells, pericytes, and Müller glial cells, promoting inflammation, apoptosis, and disruption of the blood–retinal barrier. This oxidative microenvironment plays a pivotal role in the onset and progression of diabetic retinopathy. Feedback amplification via NF-κB and other redox-sensitive signaling pathways further exacerbates oxidative stress and retinal damage.

Oxidative stress contributes to damage in neuronal, vascular, and retinal tissues, ultimately driving the development of DR (25, 26). Chronic hyperglycemia induces oxidative stress primarily through pathways involving polyol, PKC, hexosamine, and the formation of AGEs (**Figure 3**) (27).

### 3.1 Hyperglycemia-activated polyol pathway

Persistent hyperglycemia activates the polyol pathway, a key contributor to oxidative stress in the diabetic retina (28). In this process, aldose reductase (AR), the key regulatory enzyme, catalyzes the reduction of glucose to sorbitol, utilizing NADPH as an electron donor. Sorbitol is subsequently converted to fructose by sorbitol dehydrogenase, during which NAD+ is reduced to NADH (29).

Oxidative stress in DR is influenced by several factors. First, sorbitol, a highly hydrophilic alcohol, cannot diffuse through lipid membranes. Its accumulation raises cellular osmotic pressure, causing damage to retinal capillaries and leading to cell death. Second, fructose produced via the polyol pathway can be converted to fructose-3-phosphate, which is then further metabolized to 3-deoxyglucosone. Both of these metabolites act as precursors for AGEs through glycosylation processes. Lastly, the heightened activity of the glucose monophosphate shunt results in excessive NADPH consumption, thereby depleting the cofactor necessary for the synthesis of glutathione (GSH). This diminishes the cell’s ability to combat oxidative stress and disrupts the redox equilibrium (30). As a result, the activation of the polyol pathway under hyperglycemic conditions not only alters the osmotic balance of retinal capillary cells but also generates AGE precursors and exposes retinal cells to oxidative stress, potentially through redox imbalance and increased ROS production.

### 3.2 PKC pathway activation

The PKC pathway is crucial in the oxidative stress-induced development of DR. PKC, a serine/threonine kinase involved in signal transduction, is activated by signals from hormones, neurons, and growth factors. PKC modulates several physiological functions in retinal cells, such as retinal blood flow, endothelial permeability, leukocyte activation and adhesion (leukostasis), and the regulation of VEGF expression in retinal tissue (31). Moreover, PKC can enhance NADPH oxidase activity, thereby promoting ROS production in various vascular cells, including endothelial cells, smooth muscle cells, pericytes, and mesangial cells (32). Therefore, the PKC pathway activated by hyperglycemia exacerbates oxidative stress in retinal cells.

### 3.3 Hexosamine pathway flux

In diabetic patients, hexosamine levels are elevated in retinal tissues. Within the hexosamine pathway, glucose is initially phosphorylated to form fructose-6-phosphate, which is subsequently converted to glucosamine-6-phosphate by the enzyme fructose-6-phosphate aminotransferase (GFAT) (33). Studies have shown that the hexosamine pathway plays a role in mediating ROS-induced toxicity under hyperglycemic conditions (34, 35). Hyperglycemia-induced overproduction of mitochondrial superoxide inhibits GAPDH activity, which, in turn, activates the hexosamine pathway by increasing the influx of phosphorylated glucose (36). The elevated glucosamine produced by hexosamine pathway activation stimulates excessive ROS production in mitochondria, impairing mitochondrial respiration (37, 38). This further exacerbates oxidative stress, increases vascular permeability, and promotes angiogenesis.

### 3.4 AGEs formation

Chronic hyperglycemia promotes the nonenzymatic glycosylation of macromolecules, including proteins and lipids, resulting in the accumulation of AGEs (39). During this process, reducing sugars interact with the reactive sites of macromolecules to form unstable Schiff bases, which then rearrange into Amadori products—early intermediates of glycosylation, primarily consisting of carbonyl compounds. These intermediates undergo a series of complex chemical transformations, including oxidation and dehydration, and crosslink with macromolecules, ultimately yielding stable and irreversible AGEs (40). This process generates significant amounts of free radicals, which further promote AGE formation (41).

The nonenzymatic crosslinking of glucose (or other reducing sugars) with amino groups disrupts protein structure and function, as demonstrated by the stiffening of blood vessels resulting from the glycation of collagen and elastin (42). Furthermore, AGE-induced damage occurs when AGEs bind to their receptor (RAGE) on the cell surface. This binding triggers the activation of NF-κB, which leads to pericyte apoptosis in the retina and upregulation of VEGF, thereby increasing vascular endothelial permeability (43, 44). In the pathophysiology of DR, ROS production induced by AGEs plays a central role. The interaction of AGEs with RAGE activates NADPH oxidases, which subsequently amplify intracellular ROS generation (45, 46). Moreover, AGEs contribute to the increased expression of RAGE. Some studies suggest that AGEs can stimulate ROS production through the mitochondrial electron transport chain (47). This creates a feedback loop where increased ROS further promotes AGE formation (48) and RAGE expression (49), exacerbating AGE-related damage.

### 3.5 Interaction and synergy among metabolic pathways

These hyperglycemia-induced metabolic abnormalities collectively enhance retinal ROS generation, forming a vicious cycle in which oxidative stress further amplifies these glucose-related pathways and exacerbates retinal injury in DR (50). Each pathway is interlinked through ROS or other intermediates. The ROS activation of PARP induced by hyperglycemia negatively impacts GAPDH activity. Inhibition of GAPDH further stimulates the polyol pathway and enhances intracellular AGE formation by interacting with methylglyoxal (51). It also activates PKC and nuclear factor-κB (NF-κB) signaling, while increasing flux through the hexosamine pathway (52). The GFAT enzyme in the hexosamine pathway is associated with TGF-β expression and glucose-induced PKC activity (53). Excess glucose is converted into sorbitol, which causes osmotic stress, and subsequently metabolized to fructose via the polyol pathway. Byproducts of this pathway, including fructose-3-phosphate and 3-deoxyglucosone, are strong glycosylating agents that promote AGE formation (54). Ultimately, the accumulation of AGEs exacerbates oxidative stress and further activates the PKC pathway (55).

In addition to the canonical mechanisms involving mitochondrial dysfunction and NADPH oxidase activation, recent studies have expanded our understanding of oxidative stress in DR. For instance, single-cell transcriptomic profiling has revealed cell-type specific ROS responses in retinal endothelial cells and Müller glia under diabetic conditions (56). Moreover, emerging data suggest that redox-sensitive transcription factors such as NRF2 and FOXO3a play dual roles in modulating both oxidative and inflammatory pathways (57). These advances emphasize the complex regulatory networks through which oxidative stress operates in DR and provide potential new targets for intervention.

## 4 Pathological impacts of oxidative stress in DR

Oxidative stress does not act in isolation but profoundly reshapes the epigenetic landscape of retinal cells. DNA methylation, histone modifications, and non-coding RNA networks dynamically regulate gene expression in response to sustained ROS exposure (58). These changes contribute to metabolic memory, inflammation, and mitochondrial dysfunction, thereby reinforcing and perpetuating retinal damage. Importantly, oxidative stress and epigenetic dysregulation intersect with immune activation and glucose toxicity, establishing a complex and self-amplifying network of pathological signals. Understanding this crosstalk is essential for identifying novel therapeutic targets and biomarkers in DR.

### 4.1 Mitochondrial vulnerability to oxidative stress

The retina is a complex neurovascular tissue composed of multiple interacting cell types, including neurons (such as retinal ganglion cells and photoreceptors), glial cells (notably Müller cells and astrocytes), and vascular cells (endothelial cells and pericytes) (59). While oxidative stress affects all retinal cell populations, the relative timing and vulnerability remain an area of active investigation. There is ongoing debate regarding whether neuronal or vascular injury occurs first in the course of DR (60). Mitochondria are primary targets of oxidative stress in the diabetic retina. Under hyperglycemic conditions, excessive ROS production in the retina leads to mitochondrial dysfunction. mtDNA contains a large non-coding region, the displacement loop (D-loop), which houses essential transcription elements. This unwound region is highly vulnerable and plays a critical role in mtDNA replication (61, 62). Although apoptosis of retinal vascular cells has been well documented as an early hallmark of DR, accumulating evidence suggests that neuronal cells may be affected at even earlier stages (63). Studies have reported increased oxidative damage and functional deficits in retinal ganglion cells (RGCs) and photoreceptors in diabetic models prior to observable vascular abnormalities (64). Neuronal loss, glial activation, and impaired synaptic transmission have also been noted in preclinical studies and early-stage clinical imaging. These findings support the concept that DR is not only a vascular disorder but also a neurodegenerative disease from its earliest stages. In diabetes, the D-loop is more prone to damage and sequence variations than other mtDNA regions, and its copy number decreases (65). Furthermore, hyperglycemia-induced hypermethylation of mtDNA in DR impairs its transcription, contributing to mitochondrial dysfunction and subsequent capillary cell apoptosis (66, 67). Epigenetic modifications of mtDNA have been identified as a key factor in base mismatches during DR pathogenesis (68). Unlike nuclear DNA, circular mtDNA lacks protective histones, making it more susceptible to oxidative stress-induced damage (69). Damaged mtDNA disrupts transcription and protein synthesis, further impairing electron transport and exacerbating ROS production.

As mentioned above, while it is well established that retinal mitochondria are damaged in the pathogenesis of DR, the precise mechanisms by which the diabetic milieu causes this damage remain unclear. Studies have shown that, although ROS levels increase early in the retina and its capillary cells in diabetes, mitochondrial damage occurs subsequently (70). While mitochondria are the primary source of ROS, a significant amount is also generated through electron transfer from NADPH to molecular oxygen. Additionally, Nox2 activation in retinal vascular cells contributes to ROS production (71). Elevated ROS can damage mitochondrial membranes, directly opening mtKATP channels, which reduces mitochondrial membrane potential and uncouples mitochondrial respiration (72). Furthermore, Nox2 activation has been identified as an early event in DR, with cytosolic ROS progressively damaging mitochondria, leading to their dysfunction (73).

An additional mechanism contributing to mitochondrial damage in DR is the activation of gelatin matrix metalloproteinases (MMPs). Diabetes induces the activation of the NADPH oxidase (Nox) complex (74), which amplifies oxidative stress and promotes the upregulation of MMP expression (75). Both hyperglycemic conditions and oxidative stress facilitate the translocation of MMPs into the mitochondria (76). Once inside the mitochondria, MMPs degrade connexin 43, causing damage to the mitochondrial membrane and increasing its permeability (77). This damage leads to mitochondrial swelling in the retinas of diabetic mice (78) and promotes the release of cytochrome c (Cyt c) into the cytosol, which initiates the formation of the apoptosome and triggers the caspase cascade (79, 80).

### 4.2 ROS-induced retinal cell apoptosis and inflammatory pathways

While the previous section discussed mitochondrial susceptibility to oxidative damage, this section explores how such oxidative injury propagates downstream effects, particularly retinal cell apoptosis and inflammatory signaling pathways. These processes play critical roles in the early development and progression of DR. Apoptosis of retinal cells occurs early in DR, with accelerated capillary cell death detectable even before the appearance of histopathological changes (81). High glucose levels in retinal endothelial and pericyte cells exacerbate oxidative stress, leading to elevated Caspase-3 activity and activation of NF-κB and other transcription factors, further promoting capillary cell apoptosis.

Caspases, a group of cysteine proteases crucial for apoptosis, are highly sensitive to oxidative stress (82, 83). The accumulation of ROS induced by hyperglycemia increases mitochondrial membrane permeability (84), resulting in the release of cytochrome c and other pro-apoptotic factors. This process triggers apoptosis via caspase activation (85, 86). During the early stages of apoptosis, cytochrome c is detected in retinal capillary cells (87), where it activates Caspase-9, which in turn triggers Caspase-3 activation, ultimately leading to DNA fragmentation (88). Hyperactivation of Caspase-3 is frequently observed in DR, with oxidative stress playing a pivotal role in its activation. Inhibition of Caspase-3 activation has been shown to mitigate the oxidative stress-driven development of retinopathy in diabetic rats (89).

Another important factor is NF-κB, whose activation represents a critical proinflammatory and proapoptotic signaling pathway involved in hyperglycemia-induced inflammation and cellular apoptosis (90). In diabetes, NF-κB is activated early during the development of DR, and its activity remains elevated even as apoptosis of retinal capillary cells accelerates (91). As a redox-sensitive nuclear transcription factor, NF-κB plays a central role in regulating inflammatory responses and repressing antioxidant enzymes (92). NF-κB has been shown to trigger proapoptotic processes in retinal pericytes under high glucose stress, which may account for the early onset of pericyte death in DR (93). Activation of NF-κB also induces the upregulation of matrix metalloproteinase-9 (MMP-9) (94) and elevated mitochondrial MMP-9 levels cause damage to connexin-43, a mitochondrial gap junction protein, thereby increasing mitochondrial membrane permeability. This promotes the release of Cyt c into the cytosol. Additionally, the interaction between NF-κB signaling and ROS is commonly observed in cells, with these two pathways reciprocally regulating each other to facilitate inflammation and apoptosis (95).

### 4.3 Lipid peroxidation

Oxygen-derived free radicals, including hydroxyl and hydroperoxyl species, are known to oxidize phospholipids and other cellular lipids, resulting in lipid peroxidation. Retinal tissues affected by oxidative stress-related diseases exhibit elevated levels of lipid peroxidation products, underscoring the link between oxidative stress and lipid peroxidation (96). Oxidative stress induced by hyperglycemia plays a critical role in the enhancement of lipid peroxidation in DR (97). Research has shown that intracameral injection of H2O2 into the rabbit retina leads to increased lipid peroxidation in the iris epithelial cell membranes (98). Additionally, lipid metabolism contributes to the generation of oxidants, and lipid peroxidation, in turn, produces ROS, such as H2O2, which accelerates retinal pigment epithelium (RPE) cell senescence, further advancing DR. Furthermore, lipid peroxidation products facilitate the leakage of ROS from mitochondria (99).

Lipid peroxidation damages cell membranes and generates diffusible aldehydic by-products, including 4-hydroxynonenal (HNE) and 4-hydroxyhexenal (HHE). In a rat DR model, HNE has been shown to activate the canonical WNT pathway via oxidative stress, contributing to DR pathogenesis (100). HNE also promotes p53-mediated apoptosis in RPE cells (101). At femtomolar concentrations, HHE can induce the formation of mitochondrial permeability transition pores and modulate NF-κB activation, triggering various proinflammatory genes (102). Moreover, lipid peroxidation plays a significant role in the progression of neurodegenerative disorders. Studies in retinal neurodegeneration models have demonstrated increased lipid peroxidation, which is accompanied by neuronal loss (103). Under hyperglycemic conditions, ROS accumulation within mitochondria directly leads to mitochondrial dysfunction. ROS-mediated mitochondrial defects contribute to the buildup of lipid droplets in glial cells, which are further peroxidized by ROS, initiating neurodegeneration in the retina (104).

## 5 Oxidative stress and epigenetic modifications in DR

**Figure 4:**
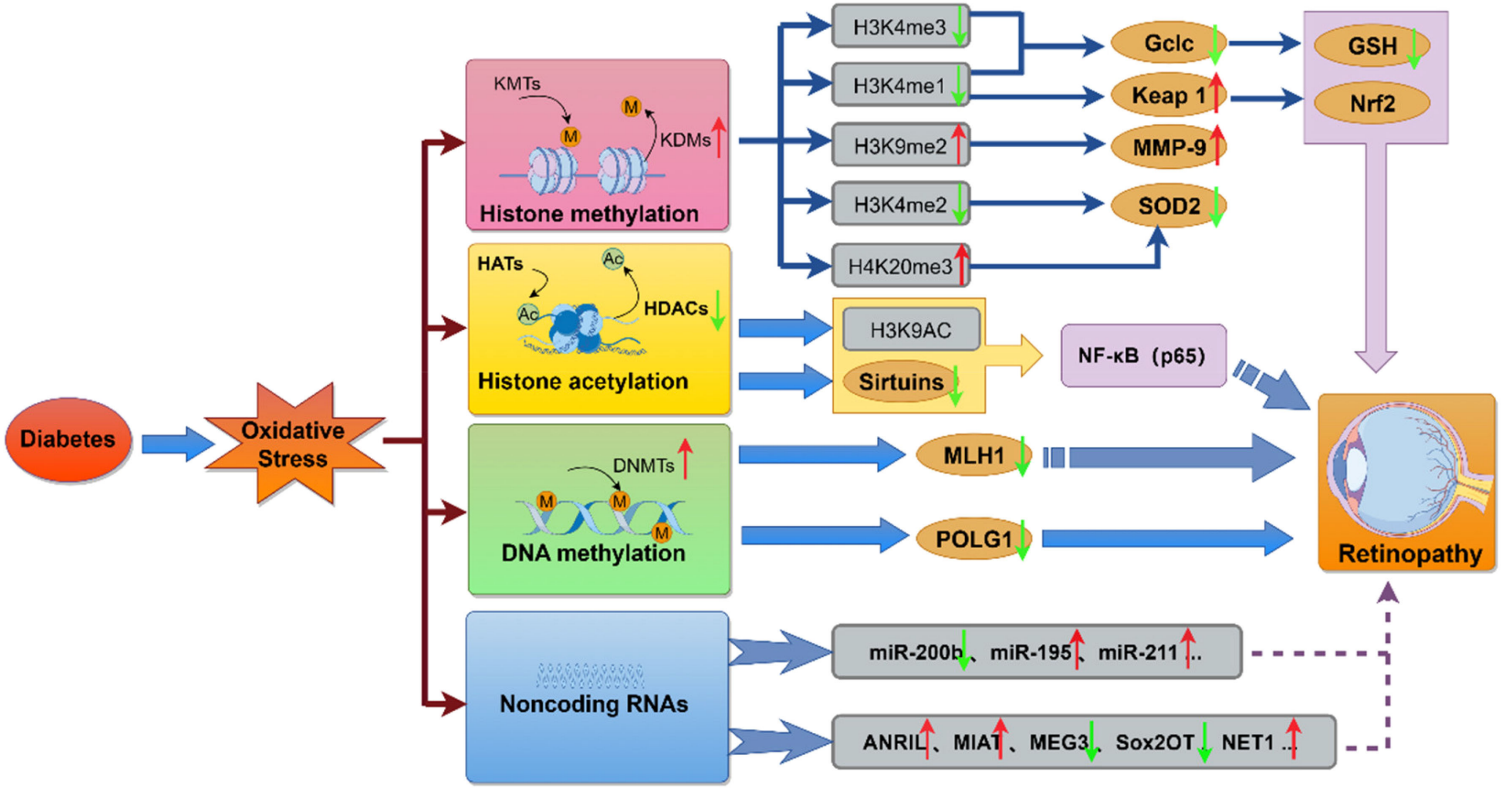
The molecular mechanisms linking diabetes-induced oxidative stress to retinopathy. This diagram illustrates how diabetes-induced oxidative stress modulates key molecular pathways, including histone methylation, histone acetylation, DNA methylation, and the regulation of miRNAs and lncRNAs. These epigenetic alterations and non-coding RNA expressions contribute to metabolic, functional, and physiological abnormalities, ultimately promoting the development of retinopathy.

Long-term hyperglycemia triggers epigenetic modifications, which, without altering the DNA sequence, regulate gene expression through mechanisms like DNA methylation, histone modification, and noncoding RNA expression (105). These changes sustain oxidative stress and inflammation, even after glycemic normalization, and are central to the phenomenon of metabolic memory in DR (106). Abnormal epigenetic alterations are closely linked to oxidative stress, as key enzymes involved in DNA methylation and histone modifications are redox-sensitive. In DR, the dysregulation of genes associated with oxidative stress is driven by hyperglycemia-induced epigenetic changes. Hyperglycemia disrupts histone and transcription factor modifications, as well as the expression of regulatory non-coding RNAs, leading to the overexpression of proinflammatory proteins and the suppression of ROS-scavenging genes (107). Additionally, genetic polymorphisms in histone methyltransferases, which promote the overexpression of proinflammatory factors involved in vascular damage, have been shown to predict the risk of both microvascular and macrovascular diabetic complications (**Figure 4**) (108).

### 5.1 Histone modification of oxidative stress-related genes

Nucleosomes are composed of repeating tetrameric units made up of histones H2A, H2B, H3, and H4, with the N-terminal regions of these histones available for posttranslational modifications such as acetylation and methylation. These modifications influence chromatin structure and regulate gene expression by affecting the binding of transcription factors to their cis-regulatory elements. Histone acetylation generally enhances gene expression and is controlled by a balance between histone acetyltransferases (HATs) and histone deacetylases (HDACs). Disruption of this balance is linked to various pathological conditions, including inflammatory and degenerative diseases (109). Another significant modification is histone methylation, where methyltransferases add a methyl group to lysine residues, while demethylases remove it. Unlike acetylation, methylation can either suppress or activate gene expression, depending on the site and degree of methylation.

In DR, most histone modifications serve as negative regulators of the antioxidant defense system in retinal cells. Epigenetic alterations induced by hyperglycemia, including increased methylation at H4K20, acetylation at H3K9, and LSD1-mediated demethylation at H3K4, affect the promoter and enhancer regions of SOD2, resulting in its downregulation. SOD2, a mitochondrial antioxidant enzyme, plays a pivotal role in mitigating oxidative stress by catalyzing the dismutation of superoxide anions into hydrogen peroxide, thus protecting cells from oxidative damage (110). In the context of DR, SOD2 has been identified as a critical player in maintaining retinal health under hyperglycemic conditions. Recent studies have highlighted the connection between SOD2 activity and metabolic memory in the pathogenesis of DR (111). Prolonged exposure to hyperglycemia has been shown to lead to epigenetic changes that downregulate the expression of SOD2 in retinal cells, resulting in the accumulation of oxidative damage that persists even after blood glucose levels are controlled. These changes in SOD2 expression are believed to contribute to the development and progression of DR, particularly by enhancing oxidative stress, mitochondrial dysfunction, and inflammatory responses in retinal tissues (112). Notably, studies in diabetic animal models have demonstrated that restoring SOD2 activity can significantly reduce oxidative damage, improve mitochondrial function, and alleviate the pathological features of DR, including retinal cell apoptosis and neovascularization (113). These findings underscore the potential of targeting SOD2 as a therapeutic strategy to counteract the persistent effects of metabolic memory in DR, offering hope for more effective interventions in diabetic patients with long-standing hyperglycemia (114). Research has demonstrated that transient hyperglycemia in aortic endothelial cells induces lasting epigenetic changes, such as mono-methylation of histone 3 at the NF-κB p65 promoter, both *in vitro* and *in vivo*. This modification enhances p65 transcription, promoting retinal cell apoptosis in diabetes (115). Furthermore, hyperglycemia-induced upregulation of retinal MMP-9 is associated with hypermethylation of H3K9 at the MMP-9 promoter, mediated by LSD1. This modification promotes MMP-9 transcription, damages mitochondria, and accelerates capillary cell apoptosis (116). In addition, oxidative stress induced by hyperglycemia modulates sirtuin-mediated epigenetic modifications by affecting sirtuin expression and activity (117). Sirtuins, a family of NAD+-dependent deacetylases, regulate protein acetylation, metabolism, and aging-related processes. Among them, SIRT1 is particularly sensitive to redox changes (118). By deacetylating transcription factors or histones, SIRT1 influences various cellular processes, including inflammation, apoptosis, and cell proliferation, thereby protecting retinal cells from oxidative stress.

### 5.2 DNA methylation

The transcriptional regulation of redox-related genes in the retina is influenced by the methylation of cytosine residues in DNA, a critical epigenetic mechanism that responds to environmental changes (119). DNA methyltransferases (DNMTs) add a methyl group to the C5 position of cytosine, resulting in 5-methylcytosine (5mC). This modification compacts chromatin, preventing transcription factors from binding. The DNMT family includes five members, with DNMT1, DNMT3a, and DNMT3b being catalytically active. DNMT1 has been found in the mitochondrial matrix, where it binds to mtDNA and regulates mitochondrial gene transcription in a targeted manner. Abnormal expression of mtDNMT1 disrupts mtDNA gene expression asymmetrically (120). In DR, DNMT1 levels are significantly elevated in mitochondria, leading to mtDNA hypermethylation, which impairs transcription and disrupts mitochondrial function, including the electron transport chain (ETC) and overall mitochondrial homeostasis (67). Additionally, hyperglycemia-induced DNMT activity increases the methylation of the POLG regulatory region, blocking its interaction with the D-loop of mtDNA, thus hindering mtDNA biogenesis (121). Changes in DNA methylation at the MMP-9 promoter in DR further upregulate MMP-9 expression. This accumulation of MMP-9 in mitochondria damages the mitochondrial membrane, triggering apoptotic pathways and contributing to the progression of DR (122, 123).

### 5.3 Noncoding RNAs

Noncoding RNAs, which do not have an open reading frame for translation, include microRNAs (miRNAs, less than 20 base pairs), long noncoding RNAs (lncRNAs, more than 200 base pairs), transfer RNAs, and circular RNAs. These noncoding RNAs can bind to the coding regions of genes or recruit proteins to modify histones, thereby altering gene expression (124).

miRNAs regulate gene expression by suppressing post-transcriptional processes through binding to target mRNAs. SIRT1, an antioxidant, inhibits NF-κB pathways and protects mitochondria (125). Studies have shown that in retinal microvascular endothelial cells exposed to high glucose, the upregulation of miR-195 accelerates the degradation of SIRT1 mRNA (126). Similarly, miR-211, which is elevated in DR patients and could serve as a biomarker, also directly targets and downregulates SIRT1 expression (127). Oxidative stress further enhances miR-195 expression, exacerbating SIRT1 suppression (128). These studies highlight the critical role of miRNAs in DR development, making them potential therapeutic targets for DR management.

Long noncoding RNAs (lncRNAs), which are more than 200 base pairs in length and do not code for proteins, regulate gene expression in several ways: 1) acting as decoys to block transcription factor binding and inhibit gene transcription; 2) guiding protein complexes to specific DNA regions; 3) serving as scaffolds to assemble protein complexes; 4) functioning as sponges to absorb miRNAs, thus reducing miRNA-mediated repression of target mRNAs; and 5) enhancing the transcriptional activity of nearby genes (129). Dysregulation of lncRNAs is strongly associated with the development of DR. For instance, lncRNA-ANRIL modulates VEGF expression in DR, and retinal cells from ANRIL-knockout diabetic mice exhibit significantly reduced VEGF levels and microvascular permeability compared to wild-type diabetic retinas (130). Hyperglycemia suppresses lncRNA-MEG3 expression, which normally elevates SIRT1 levels by sequestering miR-34a, thus inhibiting the NF-κB pathway and reducing apoptosis induced by hyperglycemia (131). Additionally, silencing lncRNA-Sox2OT influences high-glucose-induced oxidative stress by positively regulating Nrf2/HO-1 signaling, providing a potential therapeutic strategy for addressing retinal neurodegeneration in diabetes (132). Although the role of lncRNAs in DR was recognized later than that of miRNAs, their importance in DR pathogenesis is garnering increasing attention from researchers.

## 6 Therapeutic strategies targeting oxidative stress in DR

As discussed in the previous sections, oxidative stress plays a pivotal role in the pathogenesis of DR. Consequently, strategies aimed at targeting oxidative stress—such as inhibiting ROS production, neutralizing free radicals, or boosting the antioxidant defense system—show significant potential for managing and alleviating DR. The next section explores the effects of various antioxidant phytochemicals and pharmaceutical agents in reducing oxidative stress in DR.

### 6.1 Antioxidant compounds

While conventional antioxidants have shown limited success in clinical settings, **t**he field is witnessing a shift toward multifunctional antioxidant platforms, including bioactive nutraceuticals (e.g., resveratrol, curcumin, quercetin) and delivery-optimized nanoformulations (133). These agents not only scavenge ROS but also regulate redox-sensitive gene expression and mitochondrial biogenesis. The combination of antioxidant efficacy with targeted delivery holds promise for overcoming pharmacokinetic limitations and improving clinical outcomes in DR. Recent advances in formulation technology and sustained-release strategies further underscore their translational potential (134). Recent studies have highlighted the therapeutic promise of various nutraceutical compounds with antioxidant properties in DR. Among these, resveratrol, a polyphenol found in grapes, has been shown to activate SIRT1 and inhibit NF-κB signaling, thereby reducing inflammation and oxidative stress (135). Curcumin, derived from turmeric, exerts similar effects by activating the Nrf2 pathway and stabilizing mitochondrial function. Quercetin, lutein, and astaxanthin have also demonstrated protective effects in both *in vitro* and *in vivo* models of DR (136).

In addition to their intrinsic antioxidant capacity, the efficacy of these compounds has been significantly enhanced by modern drug delivery systems, such as liposomes, micelles, and nanoformulations (137). These approaches improve bioavailability, stability, and retinal targeting, overcoming major limitations in conventional administration. Several recent reviews have summarized the progress in this field (138, 139), indicating a growing interest in combining nutraceuticals with to develop more effective antioxidant therapies. Polyphenols are widely recognized for their health benefits, including antioxidant, anti-inflammatory, and anticancer effects. Their therapeutic potential in modulating oxidative stress in DR has been well explored. Green tea is rich in polyphenols, with epigallocatechin-3-gallate (EGCG) being the key compound responsible for its potent antioxidant properties. EGCG not only reduces retinal reactive oxygen species (ROS) but also exhibits neuroprotective effects, improving hyperglycemia-induced damage to the blood-retinal barrier and alleviating electroretinogram abnormalities (140). Quercetin, a flavonoid found in fruits, vegetables, and grains, boosts the expression of antioxidant enzymes, including glutathione (GSH), superoxide dismutase (SOD), and catalase in the retinas of diabetic rats. It also inhibits the expression of NF-κB and Caspase-3, effectively preventing retinal neurodegeneration and oxidative stress damage induced by diabetes (89). Resveratrol, a non-flavonoid polyphenol, is a potent scavenger of superoxide anions and singlet oxygen. It has protective effects against age-related ocular diseases, such as glaucoma, DR, and macular degeneration (141). By activating the AMPK/SIRT1/PGC-1α pathway, resveratrol helps eliminate intracellular ROS induced by hyperglycemia and prevents ROS-induced apoptosis in retinal capillary endothelial cells (142). Curcumin, an active compound in Curcuma longa, shows therapeutic potential in DR prevention through its hypoglycemic, antioxidant, and anti-inflammatory properties (143). It helps prevent structural degeneration and reduces capillary basement membrane thickening in DR (144).

Lutein, a carotenoid, is particularly effective in neutralizing singlet oxygen. In retinal tissues, lutein is concentrated in the macula, where it absorbs high-energy blue light and protects photoreceptors from phototoxic and oxidative damage (145). Additionally, lutein protects the inner retina from ischemia-reperfusion injury and inhibits oxidative stress-induced apoptosis in retinal ganglion cells and retinal pigment epithelial cells by scavenging endogenous ROS (146). Astaxanthin, a xanthophyll carotenoid known for its potent antioxidant activity, has been shown to protect retinal cells from oxidative stress. It reduces inflammation, ROS, and apoptosis by modulating various oxidative stress pathways (147). Zeaxanthin, a carotenoid found specifically in the retina, helps alleviate retinal oxidative stress and inflammation in diabetic rats (148). Lipoic acid (LA), a natural thiol antioxidant, increases cellular GSH levels by triggering the Nrf2-Gclc-GSH cascade. LA treatment boosts nuclear Nrf2 levels, facilitating the binding of Nrf2 to the antioxidant response element (ARE) to activate the transcription of genes involved in antioxidant defense and detoxification (149). Vitamins C and E, which act synergistically in counteracting oxidative damage, provide antioxidant and anti-inflammatory effects in the context of DR. Supplementation with these vitamins, along with other antioxidants, normalizes retinal oxidative stress, PKC activity, and nitric oxide levels elevated by hyperglycemia, while also mitigating early microvascular damage in DR. Long-term use of these antioxidants can help prevent the progression of early-stage DR (150). Trace elements such as zinc and copper have also been shown to alleviate the severity of DR and inhibit its progression (151). Building on these findings, recent clinical trials have explored the translational potential of antioxidant therapies in human subjects with diabetes. Alpha-lipoic acid (ALA), a mitochondrial-targeted antioxidant, has been tested in a randomized controlled trial, demonstrating modest improvements in retinal nerve fiber layer thickness (152). Similarly, resveratrol has been evaluated in small-scale human studies for its capacity to reduce systemic oxidative stress and improve retinal microvascular parameters (153). Additionally, fenofibrate—although primarily used to manage dyslipidemia—has shown protective effects in DR patients in the FIELD and ACCORD-Eye trials, which are attributed in part to its anti-inflammatory and antioxidant properties independent of lipid lowering (154). These studies suggest that antioxidant-based strategies may serve as adjunctive therapies in DR, particularly when integrated with broader metabolic control.

### 6.2 Enhancers of antioxidant defense systems

The glutathione system plays a central role in defending against oxidative stress. The reduced form of GSH directly scavenges ROS and serves as a cofactor for enzymes that help eliminate ROS (155). In diabetic rats, GSH levels are reduced in the retina, including within retinal mitochondria, as well as in pericytes cultured under high glucose conditions (156). As previously discussed, the transcription of GCLC, a key enzyme in glutathione synthesis, is suppressed in the diabetic rat retina due to epigenetic modifications at its promoter (107). These results suggest that the glutathione system is impaired in its ability to effectively scavenge ROS in the diabetic retina.

Nrf2, a transcription factor, plays a central role in the cellular response to oxidative stress (157) and also exerts anti-inflammatory effects. Under normal conditions, Nrf2 is bound by KEAP1, which directs it for polyubiquitination and degradation. However, under stress, the interaction between KEAP1 and Nrf2 is disrupted, allowing Nrf2 to translocate to the nucleus. There, it heterodimerizes with other partners and binds to antioxidant response elements, transactivating protective genes such as GCLC, HO-1, GSR (encoding glutathione reductase), and SOD1 (158).

The multifunctional deacetylase SIRT1 also plays a role in protecting against DR (159). It deacetylates transcription factors like NF-κB p65 and FOXO1, reducing their activity and helping counter inflammation and oxidative stress. Additionally, SIRT1 represses the expression of p66Shc, likely reducing oxidative stress by limiting Rac1 and Nox2 activation (160). SIRT1 expression is downregulated in the diabetic mouse retina, and its overexpression has been shown to protect against the development of DR (161).

### 6.3 Inhibitors or activators for epigenetic modification

DNA methylation may play a role in disrupting the antioxidant defense system during the progression of DR. Pharmaceutical inhibition of DNA methylation has the potential to preserve redox balance and prevent the worsening of DR (162). Several dietary phenolic compounds, known for their antioxidant effects, also modulate gene expression through epigenetic mechanisms. For example, histone deacetylase (HDAC) inhibitors like trichostatin-A and valproic acid increase Nrf2 expression by enhancing histone acetylation at the Nrf2 promoter, thereby restoring the Nrf2-mediated antioxidant defense and safeguarding cells from oxidative stress damage (163).

Additionally, the upregulation of sirtuins is essential for managing DR. Resveratrol, a SIRT1 activator, prevents the acetylation of the NF-κB p65 subunit induced by diabetes, which reduces downstream MMP-9 gene transcription and protects mitochondrial integrity in the retina (164). Resveratrol also activates SIRT3, preserving mitochondrial function (165) and preventing apoptosis caused by AGEs and p53 acetylation in human vascular endothelial cells (166). Similarly, Asiatic acid enhances SIRT1 expression, reducing cell apoptosis and ROS production, while supporting mitochondrial function (167). Furthermore, vitamin D has been shown to counteract the reduction of SIRT1 caused by H2O2 in endothelial cells (168).

While numerous studies support the therapeutic potential of antioxidants in DR, their efficacy needs validation in clinical trials. In a study of DR patients, oral supplementation with antioxidants like α-lipoic acid, genistein, and vitamins for one month led to improvements in electroretinogram oscillatory potentials (169). Another study using a multi-component nutritional formula, including vitamins E, C, D3, zinc oxide, zeaxanthin, lutein, DHA, EPA, α-lipoic acid, coenzyme Q10, benfotiamine, N-acetyl cysteine, resveratrol, turmeric root extract, and green tea, showed improvements in visual function and reduced serum inflammatory markers in diabetic individuals. These promising findings support the potential benefits of these antioxidants (170). However, clinical outcomes may be influenced by factors such as the duration and dosage of antioxidant treatment, as well as the ability of antioxidants to cross the blood-retinal barrier (BRB), which may limit their effectiveness. Despite these challenges, the encouraging results from animal studies and preliminary clinical data suggest that further investigation is necessary. Of course, beyond antioxidant-specific interventions, it is essential to address the upstream metabolic trigger—chronic hyperglycemia. Glycemic control remains the cornerstone of DR prevention and treatment, as sustained high glucose levels directly contribute to excessive ROS generation through mitochondrial overload and activation of multiple pro-oxidative pathways (138). Intensive glycemic management can suppress ROS production and delay the onset of microvascular complications. Emerging evidence also highlights the pleiotropic antioxidant effects of several antidiabetic agents. Metformin, for example, activates AMP-activated protein kinase (AMPK), enhances mitochondrial biogenesis, and restores redox balance. GLP-1 receptor agonists (e.g., liraglutide) and SGLT2 inhibitors (e.g., empagliflozin) have shown the ability to reduce oxidative stress and improve endothelial function in diabetic models and early clinical studies (171). These agents offer dual benefits—addressing metabolic dysfunction and mitigating oxidative stress—thus representing promising components of integrated DR management strategies.

## 7 Conclusion

The aim of this review is to deepen our understanding of the molecular mechanisms and pathogenic roles of oxidative stress in DR. As a prevalent and significant complication of diabetes, DR imposes a considerable socioeconomic burden. Oxidative stress, resulting from the imbalance between ROS production and their removal, is a key driver of DR’s pathophysiology. Gaining insight into the molecular underpinnings of oxidative stress is crucial for developing effective therapeutic strategies.

We have discussed how metabolic abnormalities induced by hyperglycemia, including the activation of the polyol pathway, AGE formation, PKC activation, and the hexosamine pathway, contribute to ROS production and exacerbate oxidative stress within the retina. This oxidative stress perpetuates metabolic dysfunction, creating a vicious cycle that results in mitochondrial damage, retinal cell apoptosis, lipid peroxidation, epigenetic alterations, and subsequent retinal structural and functional damage.

This review emphasizes the interconnectedness of oxidative stress with chromatin modifications, non-coding RNA networks, and the broader cellular microenvironment. Moreover, we highlight recent advances in antioxidant therapies, including bioactive nutraceuticals and nanotechnology-based delivery systems, which hold promise for improving treatment specificity and efficacy.

Future research should prioritize mechanistic studies in cell-type specific models, longitudinal tracking of oxidative biomarkers, and the clinical translation of targeted antioxidant strategies. A systems-level approach will be essential for developing effective, multifaceted interventions for DR.
